# Comparison of Children With Onset of Juvenile Dermatomyositis Symptoms Before or After Their Fifth Birthday in a UK and Ireland Juvenile Dermatomyositis Cohort Study

**DOI:** 10.1002/acr.21753

**Published:** 2012-10-27

**Authors:** N Martin, P Krol, S Smith, L Beard, C A Pilkington, J Davidson, L R Wedderburn, on behalf of The Juvenile Dermatomyositis Research Group (JDRG)

**Affiliations:** 1 Great Ormond Street HospitalLondon and Yorkhill HospitalGlasgow, UK; 2 Charles University, 1st Medical SchoolPrague, Czech Republic; 3 University College LondonLondon, UK; 4 Great Ormond Street Hospital and University College LondonLondon, UK; 5 Royal Hospital for Sick ChildrenGlasgow, UK

## Abstract

**Objective:**

To compare 2 groups of children with juvenile dermatomyositis (DM), those with onset of symptoms before their fifth birthday versus those whose disease begins either on or after their fifth birthday, and to assess whether age at onset is associated with differences in disease presentation, treatments received, or outcomes 2 years after diagnosis.

**Methods:**

Data were analyzed on children recruited to a UK juvenile DM cohort study with a diagnosis of probable or definite juvenile DM and less than 12 months between diagnosis and recruitment.

**Results:**

Fifty-five (35%) of 157 children had onset of symptoms before their fifth birthday. At diagnosis, cutaneous ulceration was found in 32.7% of the younger group versus 11.8% of the older group (*P* = 0.003). Facial or body swelling was reported more often in the younger group, whereas headaches, alopecia, and Raynaud's phenomenon were all more frequently reported in the older group. At followup 2 years later, there were no important differences in outcomes between the groups. More than 90% of patients in both groups received both methotrexate and steroids. Twenty-three percent of both groups remained on steroids 2 years after diagnosis.

**Conclusion:**

Our study showed that children with juvenile DM with disease onset at age <5 years are more likely to present with ulcerative skin disease and edema. There were no clinically significant differences in outcomes between the 2 groups.

## INTRODUCTION

Juvenile dermatomyositis (DM) is the most common of the childhood idiopathic inflammatory myopathies. It has an incidence of approximately 2–3 per million per year, with some differences between ethnic groups (1–3). Despite advances in the treatment of juvenile DM, including increasing choices in drug therapies and the use of biologic drugs, significant morbidity still occurs in a proportion of patients, particularly those with a chronic course whose disease is difficult to control (4–7). Recent surveys of pediatric rheumatologists in both the UK and North America suggest that a combination of steroids and methotrexate forms the mainstay of first-line therapy for the majority of patients with juvenile DM (8, 9). However, there is less consensus regarding treatments for those patients with an inadequate response to methotrexate and steroids. Identification of factors associated with severe disease and chronic course, to allow early choice of aggressive treatment, would represent important progress.

Expert opinion has previously suggested that a young age at onset may be associated with more severe disease (10). If true, this may be due to age-related variation in the maturing immune system or differences in exposure to environmental pathogens between children in preschool and those who have started school. A survey of patients with juvenile DM taken from a large North American registry found an increased history of febrile illness and symptoms suggestive of respiratory infection in the 3 months preceding onset of juvenile DM symptoms for patients ages <6 years. The same study found an increased frequency in the reporting of headaches preceding the onset of juvenile DM symptoms in older children (11). A recent large retrospective study with data from patients in 27 centers across Europe and Latin America compared children ages ≤5 years at onset of juvenile DM symptoms with children ages >5 years at onset and did not find early age at onset to be predictive of poor outcome (7, 12). In addition, this study found little difference in the presenting manifestations of juvenile DM relating to the age at disease onset. However, the low rates of some findings, such as cutaneous ulceration, suggest the possibility of missing data due to the retrospective nature of the study. Another study found the mean age at onset of juvenile DM for patients with calcinosis to be significantly lower (5.3 years) than for patients without calcinosis (7.1 years) (13). Regardless of the lack of clear evidence, if the belief that children presenting with juvenile DM at an early age have more severe disease is widespread among experienced pediatric rheumatologists, it is also possible that younger children receive more aggressive treatment early in the course of their disease as a result.

**Significance & Innovations**

- We found that onset of juvenile dermatomyositis symptoms before age 5 years is associated with an increased incidence of ulcerative skin disease and reported edema at presentation.
- There were no clinically significant differences in outcomes at 24 months of followup between the 2 groups.

The aim of this study was to explore whether patients with onset of symptoms prior to their fifth birthday have differences from patients with an older age at onset, either in reported symptoms and clinical findings at disease presentation, treatments received during the course of their illness, or reported symptoms and findings 2 years after diagnosis, using data from the UK Juvenile Dermatomyositis National Cohort and Biomarker Study and Repository (UK Juvenile DM Cohort study). Specifically, we tested the hypothesis that juvenile DM presenting before age 5 years was associated with more severe symptoms at presentation or disease course than in children whose symptoms begin on or after their fifth birthday. We chose age 5 years as a cutoff point both to enable comparison with the largest retrospective study previously published and because this is the age when children start school in the UK and are therefore exposed to a different range of environmental pathogens from preschool children.

## MATERIALS AND METHODS

### Patients

All of the children in this study were part of the UK Juvenile DM Cohort study, with a diagnosis of probable or definite juvenile DM as defined by the treating physician. This is a national cohort study and repository for childhood idiopathic inflammatory myopathies that aims to improve knowledge, facilitate research and clinical trials, and ultimately improve outcomes for these patients (14, 15). The study received full multicenter ethical approval and was carried out in accordance with the Declaration of Helsinki. Patients with myositis were recruited from 9 pediatric rheumatology centers throughout the UK. Serial clinical data were collected prospectively using standardized proformas, parallel to peripheral blood mononuclear cells, serum, genomic DNA, and biopsy material. For this analysis, only patients recruited between January 2000 and February 2010 with less than 12 months between diagnosis and recruitment to the UK Juvenile DM Cohort study and a diagnosis of probable or definite juvenile DM as determined by the treating physician were included.

### Statistical analysis

The children were divided into 2 groups according to age at disease onset. The younger group included those reporting the onset of symptoms of juvenile DM before their fifth birthday. The older group included those reporting symptoms of juvenile DM that began on or after their fifth birthday. Data were collected at entry to the cohort study and at a second time point an average of 24 months (range 21–36 months) later. Where there were multiple data entry points within that time span, the entry closest to 24 months after recruitment to the study was selected. Two standardized assessments of muscle strength and endurance were used, specifically, the Childhood Myositis Assessment Scale (CMAS) and manual muscle testing of 8 muscle groups (MMT8) (16–19). These tools have both been validated for the assessment of children with juvenile DM in children ages ≥4 years. In the case of the CMAS, the UK Juvenile DM Cohort study collected data using a scale with a maximum score of 53. The original CMAS was published by Lovell et al in 1999 (16) and had a maximum score of 51. Subsequent modifications to the original CMAS led to the development of a revised CMAS tool with a maximum score of 53, which was used by this study since data collection began in 2000. In 2004, Huber et al later published these revisions with one further change and a maximum score of 52 (19). The CMAS used by the UK Juvenile DM Cohort study differs from the most recently published version of the CMAS in only one point, whereby a child may score up to 5 points for maintaining an arm raise for 120 seconds, as in the originally published tool, whereas with the revised tool by Huber et al, the score for this maneuver can only be up to a maximum of 4 points for maintaining an arm raise for more than 60 seconds. Children scoring 53 in our tool would also by definition score 52 in the revised tool by Huber et al. In addition to clinical data, information on treatment was also analyzed, specifically, whether these children had ever received treatment with oral prednisolone, intravenous (IV) methylprednisolone, methotrexate, cyclophosphamide, IV immunoglobulin, hydroxychloroquine, cyclosporin, plasmapheresis, or anti–tumor necrosis factor (anti-TNF) agents. For certain clinical data items, specifically, calcinosis, melena, and cutaneous ulceration, the database was interrogated to assess whether these had occurred at any time from baseline to followup.

All data were analyzed in GraphPad software. Tests used for statistical significance were the Mann-Whitney test for nonparametric data and Fisher's exact test for categorical data, with *P* values less than 0.05 being considered significant.

## RESULTS

**Figure 1:**
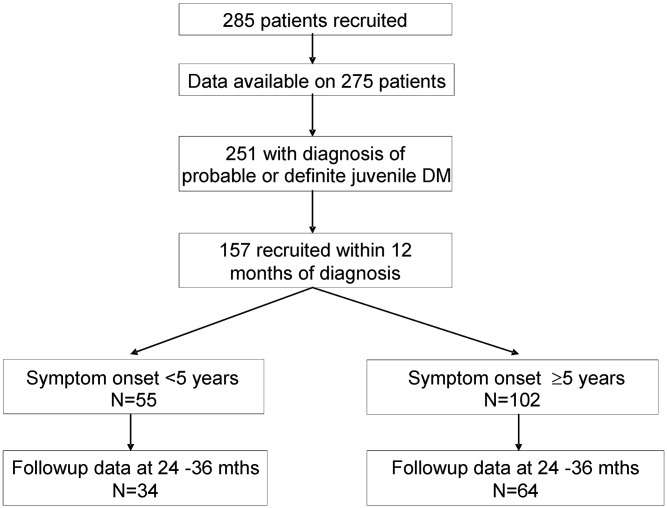
Flow chart of children included in this study and distribution into 2 groups for comparison. DM = dermatomyositis.

Of 285 cases recruited to the UK Juvenile DM Cohort study at the time of this analysis, data suitable for this analysis were available on 275, of whom 251 had a diagnosis of probable or definite juvenile DM (Figure 1). From these 251 cases, 157 had been recruited to the UK Juvenile DM Cohort study within 12 months of diagnosis and of these, 55 (35%) had developed their first symptoms before their fifth birthday, with a median age at onset of 3.1 years (range 1.2–4.9 years). The remaining 102 (65%) were in the group with symptoms presenting after their fifth birthday, with a median age at onset of 9.7 years (range 5.0–15.9 years) (Table 1). The numbers in each group with available followup data at 2 years after entry to the study were 34 (62%) in the younger group and 64 (63%) in the older group. In both groups, the median time to followup was 24 months (interquartile range [IQR] 22–25 months).

**Table 1:** Data from the initial visit at diagnosis*

	Age <5 years at onset (n = 55)	Age ≥5 years at onset (n = 102)	*P*
White ethnicity, %	78.2	77.4	1.0
Age at onset, median (range) years	3.1 (1.2–4.9)	9.7 (5.0–15.9)	
Male sex, %	38.2	25.5	0.10
Time from onset to diagnosis, median (IQR) months	4 (2–10)	4 (2–8)	1.0
Symptoms, %			
Headaches	8.9	30.3	0.005
Irritability	62.2	46.1	0.10
Alopecia	4.5	30.0	0.0006
Raynaud's phenomenon	4.0	23.4	0.047
Joint swelling	22.7	40.0	0.055
Facial/body swelling	46.5	25.8	0.028
Weight loss	31.1	37.8	0.57
Fatigue	81.8	77.8	0.66
Myalgia	65.9	71.9	0.55
Abdominal pain	26.2	20.9	0.51
Fever	27.3	21.3	0.38
Dyspnea	6.8	16.9	0.18
Dysphonia	34.9	27.0	0.42
Dysphagia	34.9	33.0	0.85
Melena	4.7	0	0.10
Examination findings, %			
Ulceration	32.7	11.8	0.003
Edema	43.4	28.0	0.07
Calcinosis	13.5	27.6	0.13
Lipoatrophy	2.9	7.3	0.65
Arthritis	22.2	37.8	0.07
Other assessments, median (IQR)			
Physicians' VAS (0–10 cm)	5.35 (2.4–7.5)	4.0 (2.2–6.8)	0.23
Parents' VAS (0–10 cm)	2.25 (0.6–4.0)	4.0 (0.9–6.6)	0.20
CMAS (range 0–53)†	19 (8–40)	41 (26–50)	N/A
C-HAQ (range 0–3)	1.563 (0.875–2.375)	1.375 (0.375–2.375)	0.36
MMT8 (range 0–80)†	43.5 (29–67)	65 (44–76)	N/A

* IQR = interquartile range; VAS = visual analog scale; CMAS = Childhood Myositis Assessment Scale; N/A = not applicable; C-HAQ = Childhood Health Assessment Questionnaire; MMT8 = manual muscle testing of 8 muscle groups.

† Both the CMAS and MMT8 have been validated only in children ages ≥4 years.

The 2 groups showed no significant differences in ethnicity, and the median time from reported symptom onset to diagnosis was 4 months in both groups. Of the younger group, 38.2% were male as opposed to 25.5% of the older group, but this difference did not reach statistical significance (*P* = 0.10).

Table 1 shows the clinical symptoms reported and examination findings at diagnosis. On clinical examination at presentation, a significantly higher rate of ulcerative skin disease was found in the younger group. Of the younger group, 32.7% were found to have ulceration versus 11.8% of the older group (*P* = 0.003). The mean age at onset for patients with ulceration at diagnosis was 5.7 years versus 7.6 years for patients without ulceration at diagnosis (*P* = 0.019). There were trends for less arthritis and more edema found on examination in the younger children, but these did not reach significance. There were no significant differences in the rates of lipoatrophy or calcinosis found on initial examination.

In the analysis of symptoms reported, there were significant differences in the rate of 4 clinical items analyzed: headaches, alopecia, and Raynaud's phenomenon were all reported more frequently in the older group than in the younger group, whereas facial or body swelling was reported by patients more often in the younger group than in the older group. Parallel to findings on clinical examination, there was a trend toward increased joint swelling reported in the clinical history among the older group. There were no significant age-related differences in the frequency of other symptoms reported at presentation, including weight loss, fatigue, myalgia, abdominal pain, fever, dyspnea, dysphonia, dysphagia, and melena.

Disease severity and functional impairment at presentation as measured by parent and physician visual analog scales and the Childhood Health Assessment Questionnaire (C-HAQ) were not significantly different between the 2 groups. The assessment of muscle strength at the initial visit using both the CMAS and MMT8 has been included to demonstrate the range of muscle weakness in this cohort of patients, but neither tool has been validated in children ages <4 years and therefore no comparison between these 2 groups was made using these scores.

Table 2 shows data from the second time point analyzed. For both groups, followup data were collected prospectively and data from a second time point were analyzed a median of 24 months later (IQR 22–25 months). In both groups, disease activity had fallen and muscle strength had improved considerably. Twenty-three percent of children in each group were still receiving steroids at the followup visit. Median values for the C-HAQ and physician and parent 0–10-cm visual analog scales were similar at followup for both groups, with no clinically significant differences in these measures between the groups. Median values for the CMAS at followup were 50 in the younger group and 53 in the older group.

**Table 2:** Followup data at median 24 months (IQR 22–25 months)*

	Age <5 years at onset (n = 34)	Age ≥5 years at onset (n = 64)	*P*
Other assessments			
Physicians' VAS (0–10 cm)	0.2 (0–1.2)	0.45 (0–1.0)	0.62
Parents' VAS (0–10 cm)	0.0 (0–1.2)	0.05 (0–1.5)	0.96
CMAS (range 0–53)	50 (44–52)	53 (51–53)	N/A
C-HAQ (range 0–3)	0.25 (0–1.0)	0 (0–0.375)	0.16
Remaining on steroid therapy, %	23.5	23.0	1.0
Examination findings, %			
Rash	41.2	32.8	0.51
Nailfold changes	12.0	27.3	0.22
Muscle weakness	18.2	15.9	0.78
Arthritis	0	3.2	0.55
Contractures	0	8.1	0.16
Calcinosis	9.4	12.5	0.75
Ulceration	5.9	1.6	0.28

* Values are the median (interquartile range [IQR]) unless otherwise indicated. VAS = visual analog scale; CMAS = Childhood Myositis Assessment Scale; N/A = not applicable; C-HAQ = Childhood Health Assessment Questionnaire.

None of the children in the younger group had arthritis or contractures at followup, whereas 3.2% of patients in the older group had arthritis at followup and 8.1% had contractures. At followup, there was no difference between the 2 groups regarding the presence of rash. Two patients (5.9%) in the younger group were found to have skin ulceration at followup versus 1 patient (1.6%) in the older group.

In addition to data from the followup visit, data were collated for calcinosis and skin ulceration over the entire followup period to investigate if these features had ever occurred in the timeframe studied. The frequency of calcinosis overall showed no significant differences between the groups (20.6% and 18.8% in the younger and older groups, respectively), whereas the rate of skin ulceration occurring at any time during the course of their disease in the timeframe studied was higher in younger children, but this difference did not reach statistical significance (35.3% and 25%, respectively).

The frequencies of treatment with methotrexate, oral steroids, IV steroids, hydroxychloroquine, and cyclosporin were all similar for both groups. A greater proportion of children in the younger group had received cyclophosphamide or anti-TNF therapy, although this did not reach statistical significance.

## DISCUSSION

These data suggest that there are differences in the clinical features at presentation for patients with juvenile DM related to age at onset. The study revealed that children with a young age at onset are more likely to present with ulcerative skin disease and edema. Ulceration is one of the most severe cutaneous manifestations of juvenile DM and is widely considered to be an indication for aggressive medical treatment, often with IV steroids and cyclophosphamide (20, 21). Both cutaneous and gastrointestinal ulceration in juvenile DM are thought to be a result of an occlusive vasculopathy (22), and gastrointestinal vasculopathy has been associated with severity of capillary nailfold changes (23). It would be reasonable to assume a possible correlation between gastrointestinal and cutaneous ulceration. Our study was not designed to collect comprehensive data regarding endoscopic findings to confirm gastrointestinal ulceration. Documented gastrointestinal ulceration was rare. However, the only 2 patients in this cohort who reported melena at presentation were both age <5 years at the onset of symptoms and also had evidence of cutaneous ulceration at presentation. Throughout the 2-year period of followup, 2 further patients developed melena, neither of whom had cutaneous ulceration. With such small numbers, our study is unable to confirm a link between cutaneous ulceration and the presence of melena, suggestive of gastrointestinal ulceration.

Prior studies have correlated both cutaneous ulceration and chronic ulcerative disease course with direct immunofluorescence staining of intramuscular arteries, infarcts, or obvious foci of severe capillary loss on muscle biopsy (24, 25). We have previously published a proposed tool for the assessment of muscle biopsy samples in juvenile DM (26), and work to evaluate the severity of biopsy features according to age at onset, in particular within the vascular domain of pathologic change, is ongoing. In adult-onset DM, skin ulceration has been associated with specific autoantibodies such as anti–melanoma differentiation–associated protein 5 (27). In juvenile DM, it has been shown that children with an antibody to the doublet p155/p140 (of which one antigen has been shown to be the nuclear protein transcription intermediary factor 1-γ) have more extensive skin disease, including more rash, ulceration, and edema, than those who are negative for anti-p155/p140 (28). Our findings of increased skin ulceration in younger children may reflect a tendency toward prominence of a vasculitic component in the clinical presentation of children who develop juvenile DM at an early age.

The results of this study suggest that younger children are less likely to report alopecia, headaches, or Raynaud's phenomenon at presentation. When considering whether headache is truly a more common presenting feature in older children, it is of note that irritability was more frequently reported as a presenting symptom in the younger group, and these differences may represent age-related differences in the way headache is manifested and reported. The finding of more Raynaud's phenomenon and alopecia in the older group may reflect the proportion of patients with juvenile DM whose illness develops features that may overlap with another autoimmune disease such as scleroderma or systemic lupus erythematosus. Patients with juvenile DM and scleroderma overlap often present with Raynaud's phenomenon, and this is more common in older children (3).

In keeping with recent surveys of pediatric rheumatologists in both North America and the UK (8, 29), steroids and methotrexate formed the mainstay of treatment for children in this study, who are recruited from centers across the UK, with more than 90% of children in both groups receiving these drugs as part of their treatment. At followup 2 years after diagnosis, only 23% of patients from our cohort remained on steroid therapy. This is considerably lower than was found in a multicenter international trial that collected prospective data from 275 patients with juvenile DM from 36 countries between 2001 and 2004, where 52% of patients remained on steroids at followup 2 years after recruitment (29). This finding may represent differences in therapeutic approaches internationally, reflecting the lack of evidence underpinning current treatment strategies, or recent changes in treatment approaches toward a reduction in the duration of steroid therapy for patients with juvenile DM. In particular, the consistent early use of methotrexate in the UK may allow steroids to be safely reduced and stopped in most patients less than 2 years after diagnosis (30).

In our contributing centers, medications such as cyclophosphamide and anti-TNF agents are also fairly commonly prescribed in young children with juvenile DM (Table 3). Severe skin disease with ulceration is likely to have been the indication for using cyclophosphamide in some of these patients. In addition, both cyclophosphamide and anti-TNF agents are generally considered as second-line agents to be used where response to initial therapy with steroids and methotrexate is insufficient (8, 9, 20, 31). With that in mind, it is possible that young children with juvenile DM, as well as having more severe skin disease at presentation, are less responsive to standard therapy or that they receive more aggressive treatment due to a perception that they are more likely to have a poor outcome. Similarly, although there were no important differences in outcomes between the 2 groups studied here, the younger children may have received more aggressive therapy that prevented the development of morbidities such as calcinosis, weakness, or joint contractures and prevented the development of further ulceration. Unfortunately, current clinical practice in the centers contributing to this study does not include the routine use of tools to objectively quantify the severity of skin disease in juvenile DM, although several tools have been published (32–36). Therefore, the UK Juvenile DM Cohort study does not collect such data that would have been valuable in confirming the increase in the severity of skin disease at presentation in younger children suggested by the presence of ulceration. As a result, this study was also unable specifically to quantify the severity of skin disease in the large proportion of children in both groups (41.2% and 32.8% of the younger and older groups, respectively) who continue to have rash at followup 2 years after diagnosis. This limitation of our analysis highlights the importance of developing the existing available skin disease assessment tools as part of an internationally validated and accepted set of core outcome variables for patients with juvenile DM.

**Table 3:** Treatments or findings ever documented for patients with 2-year followup data*

	Age <5 years at onset (n = 34)	Age ≥5 years at onset (n = 64)	*P*
Oral steroids	94.1	93.8	1.0
IV steroids	47.1	45.3	1.0
Methotrexate	97.1	93.8	0.66
Cyclophosphamide	41.2	25.0	0.11
Anti-TNF	20.6	11.2	0.23
IVIG	8.8	12.5	0.74
Hydroxychloroquine	14.7	14.1	1.0
Cyclosporin	5.9	9.3	0.71
Plasmapheresis	2.9	1.6	1.0
Abnormal lung function	5.9	15.6	0.21
Calcinosis	20.6	18.8	1.0
Ulceration	35.3	25.0	0.35

* Values are the percentage. IV = intravenous; anti-TNF = anti–tumor necrosis factor; IVIG = IV immunoglobulin.

Proximal muscle weakness is a central feature of juvenile DM; therefore, another problem is the inability to accurately and objectively compare proximal muscle weakness between the 2 groups, since the tools used may not be accurate in the younger group. The UK Juvenile DM Cohort study collects objective serial measurements of muscle strength using the CMAS and MMT8. Where possible, these data have been collected on all children with juvenile DM. However, both of these measures have been validated only in children ages ≥4 years, and the CMAS in particular is difficult for very young children to perform. In addition, when the CMAS is used to test muscle strength and endurance in healthy children, a clear improvement in scores with increasing age has been shown. Interestingly, both groups of children have higher CMAS scores 2 years after diagnosis than healthy controls of a similar age, with the majority of children in the older group achieving a maximum score at followup. As Rennebohm et al commented, this may be because children with juvenile DM perceive that their performance in the CMAS is being used to determine their progress and medication needs. As a result, they are often highly motivated to achieve the best scores possible (17). Because neither the CMAS nor MMT8 is validated in children ages <4 years, it is not possible, with the current data set, to ascertain whether there was an objective difference in muscle weakness between the 2 groups reflecting increased severity of muscle inflammation in either group. The development and validation of a specific examination tool that was aimed at this younger group would be a valuable addition to the tools used to assess children with inflammatory myopathy.

An alternative approach to quantify the severity of muscle inflammation in children with juvenile DM would be to use an objective and validated scoring system assessing muscle inflammation using magnetic resonance imaging (MRI), perhaps in conjunction with a recently developed tool that quantifies severity of pathologic change on muscle biopsy (26). Quantifiable measures such as MRI T2 relaxation times have been shown to correlate well with other measures of muscle inflammation (37) and could be measured in patients of all ages. There is increasing evidence that in many parts of the world MRI is used more frequently than either electromyography or muscle biopsy in the initial diagnosis of juvenile DM (38), and is also often used to assess response to treatment (9). For this to be usefully implemented in a multicenter study standardization of MRI, imaging protocols and interpretation would be required. As yet, such standardized protocols are not available, although a scoring system for the assessment of active inflammation using MRI in juvenile DM has recently been proposed (39).

There are a number of differences in the findings between our study and a recently published international, multicenter, retrospective study that analyzed data from the case notes of 490 patients with juvenile DM from both Europe and Latin America (7, 12). This study by Ravelli et al, designed to assess long-term outcome and prognostic factors in juvenile DM, found no differences in presenting manifestations in children according to disease onset other than an increase in dysphagia reported by patients presenting at age >10 years. The rate of skin ulceration documented at presentation was much lower than in our cohort at 7.6% for children with disease onset before 5 years, and no significant difference was found between the age groups. Potentially, the different findings between that study and the current one may be explained by the differences in data collection methods, with information retrospectively collected from case notes in some cases more than 20 years after diagnosis. In addition to the prospective nature of the present study, it is also likely that the introduction of standardized data collection forms throughout participating centers in the UK Juvenile DM Cohort study has harmonized and focused the assessment of children with juvenile DM, making it more likely that ulceration at presentation would be identified and documented. In agreement with our findings, the study by Ravelli et al did not find that age at onset was predictive of outcome.

The strengths of this study include the relatively large number of children in the UK Juvenile DM Cohort study and the ongoing prospective nature of the data collected. However, this study also has a number of significant limitations, including the lack of a skin assessment tool to collect data regarding the severity of cutaneous disease and the inability to objectively and reproducibly assess muscle strength in children ages <4 years. Although we found no important differences in outcome between the 2 groups, some of the children included in the initial part of the study had not yet reached 2 years of followup. Therefore, the number of children analyzed in this part of the study may have been too small to show potentially important differences in outcomes. Using known data regarding the incidence of juvenile DM in the UK (2), it has been estimated that there may be a total of approximately 500 children with juvenile DM in the UK. At the time of this study, the UK Juvenile DM Cohort study had collected approximately half this number via 9 centers with specialist pediatric rheumatology services. These centers include most of the larger tertiary centers for pediatric rheumatology in the UK. As a result, this study is likely to contain a bias reflecting a high proportion of children with severe juvenile DM, whereas many children with milder disease may be cared for without these specialist centers.

In conclusion, we have shown that there are differences in the presenting features of juvenile DM in children with onset of disease at a young age when compared with children whose disease begins later in childhood. Most importantly, in our population, young age at onset was associated with more ulcerative skin disease at presentation. However, we found no evidence that a younger age at onset was associated with poor outcome at followup 2 years later. There was a trend toward the use of more intensive treatment approaches in younger children with juvenile DM. It is not clear to what extent this reflects the increase in ulcerative skin disease at presentation, or whether differences in response to therapy for younger children or a preexisting perception among physicians that younger children with juvenile DM require more intensive treatment also play a part. Our study also highlights the difficulty in objectively measuring proximal muscle weakness in young children with juvenile DM in a consistent and reproducible manner and the importance of a universally accepted tool for assessing the severity of skin disease in juvenile DM.

## AUTHOR CONTRIBUTIONS

All authors were involved in drafting the article or revising it critically for important intellectual content, and all authors approved the final version to be published. Dr. Martin had full access to all of the data in the study and takes responsibility for the integrity of the data and the accuracy of the data analysis.

**Study conception and design.** Martin, Krol, Smith, Pilkington, Davidson, Wedderburn.

**Acquisition of data.** Martin, Krol, Smith, Beard, Pilkington, Wedderburn.

**Analysis and interpretation of data.** Martin, Krol, Pilkington, Davidson, Wedderburn.
